# Molecular changes indicative of cartilage degeneration and osteoarthritis development in patients with anterior cruciate ligament injury

**DOI:** 10.1186/s12891-016-0871-8

**Published:** 2016-01-13

**Authors:** Ioanna Papathanasiou, Sotirios Michalitsis, Michael E. Hantes, Marianna Vlychou, Lydia Anastasopoulou, Konstantinos N. Malizos, Aspasia Tsezou

**Affiliations:** Laboratory of Cytogenetics and Molecular Genetics, University of Thessaly, Faculty of Medicine, Biopolis, 41500 Larissa, Greece; Department of Orthopaedic Surgery, University of Thessaly, Faculty of Medicine, Biopolis, 41500 Larissa, Greece; Department of Radiology, University of Thessaly, Faculty of Medicine, Biopolis, 41500 Larissa, Greece; Department of Biology, University of Thessaly, Faculty of Medicine, Biopolis, 41500 Larissa, Greece

**Keywords:** ACL rupture, Osteoarthritis, Inflammation, MMPs, Apoptosis

## Abstract

**Background:**

Anterior cruciate ligament (ACL) tear is considered a risk factor for osteoarthritis development. The purpose of our study was to investigate the expression levels of the apoptotic enzyme caspase 3, pro-inflammatory cytokines interleukin-1β (IL-1β) and interleukin-6 (IL-6) and degrading enzyme matrix metalloproteinase 13 (MMP-13), all indicative of cartilage degeneration and osteoarthritis development in patients’ chondrocytes after ACL rupture.

**Methods:**

We investigated the correlation between grade of cartilage degradation and time from injury or patients’ age. IL-1β, IL-6 and MMP-13 mRNA expression levels were investigated in normal (*n* = 4) and chondrocytes from patients with ACL rupture (*n* = 33) using real-time polymerase chain reaction (PCR). Moreover, MMP-13 and caspase-3 protein expression levels were evaluated by western blot analysis. Trend analysis and correlation coefficient were performed to derive the relations between gene expression (MMP13, IL-6, IL-1β) and grading of cartilage defects and between gene expression (MMP13, IL-6, IL-1β) and patients’ age, respectively.

**Results:**

Correlations were established between grade of cartilage degradation and time from injury. MMP-13, IL-6, IL-1β and caspase 3 expression levels were significantly upregulated in chondrocytes from ACL-deficient knee compared to normal. Among the patients with ACL-deficient knees, a significant upregulation of MMP-13 was observed in patients with ACL-rupture > 18 months from the time of injury to arthroscopy compared to patients with ACL-injury up to 18 months, whereas IL-6 and IL-1β expression was higher in chondrocytes from patients with more than 10 months ACL injury compared to those that underwent surgery within the first 10 months after injury. Νο association was observed between IL-1β, IL-6 and MMP-13 expression levels and cartilage defects or patients’ age.

**Conclusion:**

Our results showed that increased levels of apoptotic, inflammatory and catabolic factors in chondrocytes are associated with time from injury and could contribute to cartilage degradation and osteoarthritis development after ACL rupture.

## Background

ACL tear is a common and severe knee injury affecting mainly young and active population [1]. It has been well documented that after ACL tear more than 50 % of individuals show radiographic evidence of osteoarthritis 10–12 years post injury [2, 3]. Potential etiological factors contributing to osteoarthritis development in the ACL-deficient knee are patients’ age at time of injury, time from the onset of injury, obesity, joint malignment, concomitant presence of an acute meniscal or cartilage injury, as well as biological factors, such as increase in catabolic activity of chondrocytes, inflammatory cytokines and proteases activation in the joint after trauma [4, 5]. It is known that as a response to mechanical forces and consequent matrix loss, the healthy adult chondrocyte ‘wakes up’ from its low metabolic status and produces a series of inflammatory mediators, many of which are normally produced by macrophages in response to injury or infection. Thus, cytokines, chemokines, reactive oxygen species, prostaglandins and leukotrienes, increase the catabolic activity of the chondrocyte, resulting in the release of proteolytic enzymes, such as aggrecanases and matrix metalloproteinases, that cause destruction of cartilage matrix [6, 7]. Moreover, healthy chondrocytes produce growth factors, such as BMP-2, BMP-7, insulin-like growth factor 1, and transforming growth factor β, that stimulate matrix production and inhibit the production of proteolytic enzymes. Many of these are stored in the cartilage bound to matrix proteins and are released when the matrix is degraded so that they can act locally to shut down the degradation process [8]. In addition, apoptotic agents and their inhibitors, interact in a homeostatic equillibrium in the healthy chondrocyte, as part of the normal physiology [9].

Animal models of surgically induced osteoarthritis after ACL transection have been used to clarify the mechanisms of cartilage degradation after joint injury. Among key biological processes identified are increased extracellular matrix (ECM) turnover and remodelling, angiogenesis, chondrocytes’ apoptosis, subchondral bone plate rupture, inflammation and cholesterol metabolism, all of which are associated with human osteoarthritis pathogenesis [10, 11].

Although several cytokines have been involved in the aetiology of post-traumatic osteoarthritis, the time-course changes of cytokines’ levels after ACL injury and their contribution to cartilage degradation is not clear. It has been shown that pro-inflammatory cytokines are released in the synovial fluid in patients with acute or chronic ACL injury and are involved in MMPs stimulation and initiation of cartilage degradation [12–15]. Increased protein levels of MMP-3 and tissue inhibitor of metalloproteinases 1 (TIMP-1), as well as elevated proteoglycan and type II collagen fragments have been observed in the synovial fluid of patients following an ACL or meniscal tear from day 1 up to 20 years post injury [16, 17], suggesting an unbalanced degrading process, that can significantly increase the risk of osteoarthritis.

Besides inflammatory cytokines and degrading enzymes, apoptosis has also been demonstrated to be involved in osteoarthritis development after mechanical injury in animal models and in human joints, evidenced by the activation of caspases, enzymes that regulate and execute apoptosis [18–20]. Chondrocytes’ death, mostly through apoptotic mechanisms contributes to matrix degradation due to reduction of functional cells that are required to repair and maintain the extracellular matrix.

The purpose of the present study was to investigate molecular changes in apoptotic enzymes, inflammatory cytokines and metalloproteinases indicative of cartilage degeneration and osteoarthritis development in patients after ACL injury. For this reason, we tested the expression levels of representative molecules for the above processes, such as caspase 3, a key molecule for apoptotic mechanisms, IL-1β and IL-6, the basic inflammatory catabolic cytokines and MMP-13, the major catabolic enzyme of cartilage degradation.

## Methods

### Patients and cartilage samples

Articular cartilage samples were obtained from 33 patients undergoing knee arthroscopy following ACL rupture (mean age 25.25 years ± 8.14). Αll samples were obtained from a predetermined non-weight bearing area of the lateral femoral condyle, with the same instrument (arthroscopic spoon) in order to ensure the same sample dimension. Standard arthroscopic portals including an anterolateral and an anteromedial portal were used in all cases. A thorough evaluation of the joint was performed in every case using the anterolateral portal as viewing portal and the anteromedial portal as a working portal (using a probe). ACL rupture was assessed and confirmed arthroscopically using a probe. During ACL reconstruction, the entire articular surface was arthroscopically visualized and articular cartilage lesions were graded (0–4) according to the ICRS classification [21]. The inclusion criteria of patients were: a. ACL rupture in the affected knee established both clinically and with MRImaging, b. no previous knee surgery, c. skeletal maturity and d. no other ligamentous injury or lower limp malalignment. The exclusion criteria were as follows: (a) age more than 55 or less than 15 years, (b) previous major injury in the lower extremities and (c) rheumatic diseases or psychosocial disorders. Normal articular cartilage was obtained from 4 young healthy individuals (mean age 27.25 years ± 4.11), undergoing fracture repair surgery around the knee joint and above knee amputations after crush injuries. Written informed consent was obtained from all individuals of the study. The study protocol conformed to the ethical guidelines of the 1975 Declaration of Helsinki as reflected in a priori approval by the Local Ethical Committee of the University Hospital of Larissa.

### Primary cultures of human articular chondrocytes

Articular cartilage was dissected and subjected to sequential digestion with 1 mg/ml pronase and 1 mg/ml collagenase P (Roche Applied Science, Mannheim, Germany). Chondrocytes were counted and checked for viability using trypan blue staining. More than 95 % of the cells were viable after isolation. Isolated chondrocytes from individual specimens were separately cultured with Dulbecco’s Modified Eagles Medium/Ham’s F-12 (DMEM/F-12) (GIBCO, Life Technologies, Paisley, UK) plus 5 % fetal bovine serum (FBS, Invitrogen, Life Technologies, Paisley, UK) at 37^ο^C under a humidified 5 % CO_2_ atmosphere until reaching confluence for 4–6 days. Cultured chondrocytes were then harvested by trypsinization and were used for RNA and protein extraction.

### RNA extraction and quantification of mRNA expression

Total cellular RNA was extracted from cultured chondrocytes using Trizol reagent (Invitrogen, Life Technologies, Paisley, UK). Preservation of 28S and 18S ribosomal RNA (rRNA) species was used to assess RNA integrity. All the samples included the study were with prominent 28S and 18S rRNA components. The yield was quantified spectrophotometrically. Transcription of 1 μg RNA to cDNA was performed using SuperScript III reverse transcriptase (Invitrogen, Life Technologies, Paisley, UK) and random primers (Invitrogen, Life Technologies, Paisley, UK). Quantification of MMP-13, IL-6 and IL-1β mRNA expression was performed by real-time PCR (ABI 7300, Applied Biosystems, Foster, CA). The oligonucleotide primers used for MMP-13, IL-6 and IL-1β amplification are showed in Table 1. To quantify the relative expression of each gene, Ct values were normalized against the endogenous reference (ΔCt  =  Ct target – Ct GAPDH) and were compared with a calibrator using the ΔΔCt method (ΔΔCt  =  ΔCt sample – ΔCt calibrator).

**Table 1:** Oligonucleotide primers used in real-time PCR assay

Gene	Forward primer sequence	Reverse primer sequence
MMP-13	TGGCATTGCTGACATCATGA	GCCAGAGGGCCCATCAA
IL-1β	GGGCCTCAAGGAAAAGAATC	TTCTGCTTGAGAGGTGCTGA
IL-6	ATGCAATAACCACCCCTGAC	GAGGTGCCCATGCTACATTT
GAPDH	GAGTCAACGGATTTGGTCGT	GACAAGCTTCCCGTTCTCAG

### Protein extraction and Western blot analysis

Chondrocytes were lysed using RIPA buffer containing 10 mM Tris (pH 7.5), 150 mM NaCl, 1 % Triton X-100, 1 % Sodium Deoxycholated, 0.1 % SDS, 1 mM EDTA, and a cocktail of protease inhibitors. Protein concentration was quantified using the Bio-Rad Bradford protein assay (Bio-Rad Protein Assay, BioRad, Hercules, CA, USA) with bovine serum albumen as standard. Cell lysates from chondrocytes were electrophoresed and separated on 10 % acrylamide gels and transferred to PVDF membranes (Millipore, Billerica, MA, USA). The membrane was probed with anti-MMP-13 (Abcam, Life Technologies, Paisley, UK) and anti-caspase 3 (Santa Cruz, Santa Cruz Biotechnology, CA, USA) and signal was detected using anti-rabbit immunoglobulin IgG conjugated with horseradish peroxidase (1:10000 dilution) (Invitrogen, Life Technologies, Paisley, UK). The results were normalized using anti-β-actin polyclonal antibody (1:3000 dilution) (Sigma-Aldrich, Missouri, USA). PVDF membranes were then exposed to photographic film and western blot bands from several different blots were quantified using the NIH Scion Image according to the software’s guidelines.

### Statistical analysis

Data were analysed by the non-parametric Kruskal-Wallis test using the SPPS 20 software. Where significant variance was demonstrated, differences between individual groups were then determined using Mann–Whitney 2-tailed *U* test. Trend analysis (Jonckheere test) was used to derive the relations between gene expression (MMP13, IL-6, IL-1b) and grading of cartilage defects and to estimate the significance of the relations. Correlation coefficients were calculated by Spearman rank correlation. *P*-value less than 0.05 was considered statistically significance.

## Results

### Culture experiments

All cartilage samples (33 from patients with ACL injury and 4 cartilage from healthy individuals) were successfully cultured and chondrocytes were obtained. We therefore used for all experiments cultured chondrocytes and not fresh tissue, as we have previously reported that there are no differences in genes’ and protein expression levels between cultured and chondrocytes obtained from fresh tissue [22].

All normal cartilage samples from healthy individuals (*n* = 4) and 6 cultured cartilage samples taken at random from the 33 patients with ACL injury were used as indicative ones for the detection of caspase 3 and MMP-13 protein expression by Western blot analysis. For the evaluation of IL-1β, IL-6 and MMP-13 mRNA levels, all normal cartilage samples (*n* = 4) and 28 random cartilage samples from the 33 patients with ACL injury were used. Moreover, all experiments regarding mRNA (IL-1β, IL-6 and MMP-13) and protein levels (caspase 3 and MMP-13) were evaluated at 4 different time periods (6, 10, 18 and 24 months). Accordingly, we separated all cartilage samples after ACL injury in 2 groups for each time period. More specifically;

Group A: ACL injury < 6 months and >6 months
Group B: ACL injury < 10 months and >10 months
Group C: ACL injury < 18 months and >18 months and
Group D: ACL injury < 24 months and >24 months.

### Articular cartilage damage, time from ACL injury and patients’ age

**Fig. 1:**
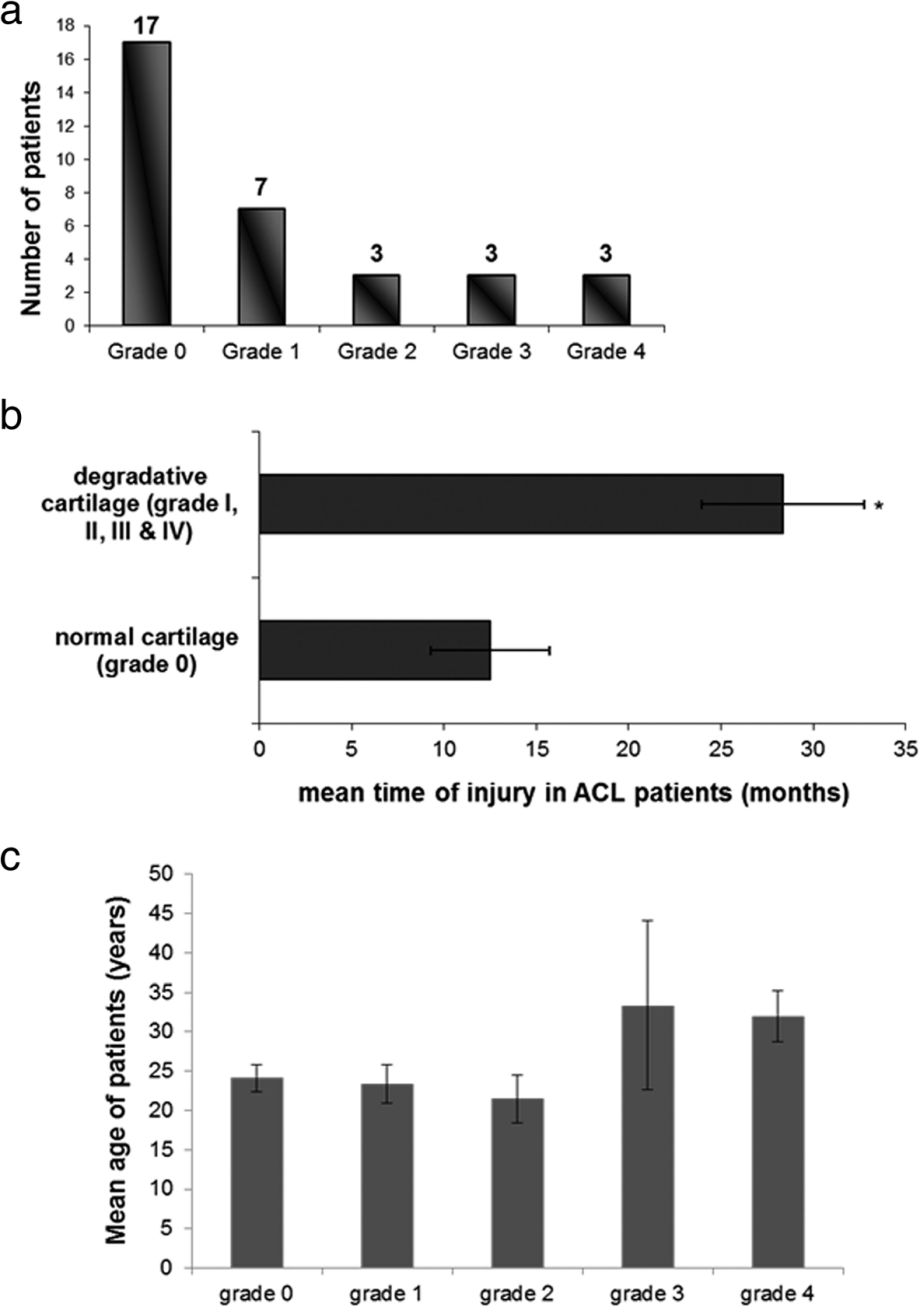
Correlation between articular cartilage damage and time from ACL injury or patients’ age. **a** The number of patients with ACL rupture based on the ICRS classification. **b** Correlation between average time from injury with cartilage damage (ICRS grade I, II, III and IV) and those with no chondral lesions (ICRS grade 0) and **c** Grade of chondral damage versus mean patients’ age

The number of patients included in the study and the ICRS grading is shown in Fig. 1a. Correlation coefficients were calculated for ACL injury to determine possible associations between grade of cartilage degradation and time from injury. Our results showed that the time from injury to arthroscopy was significantly greater in patients with damaged articular cartilage (ICRS grades I, II, III and IV) (28.36 ± 4.4 months) compared to patients with normal articular surfaces (ICRS grade 0) (12.5 ± 3.2 months) (*p* < 0.05) (Fig. 1b). Moreover, the mean age of patients in the different ICRS grades is shown in Fig. 1c. No correlation was observed between patient’s age at the time of injury and grade of cartilage damage (ICRS grade 0, I, II, III and IV).

### Caspase 3 expression in ACL-deficient knees

**Fig. 2:**
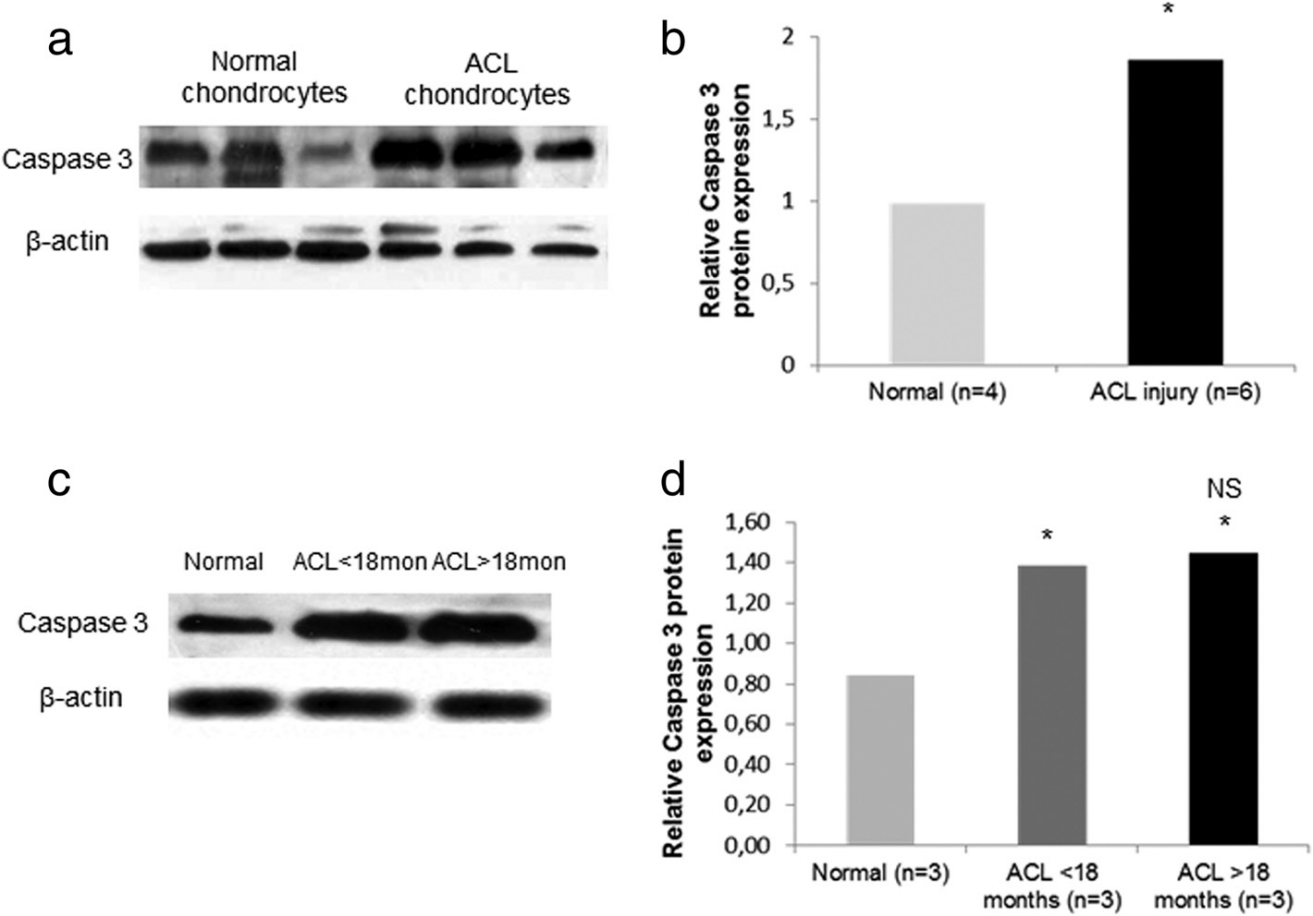
Caspase 3 expression in ACL-deficient knees. **a** and **b** Representative western blot of Caspase 3 protein expression in cultured normal chondrocytes and chondrocytes from patients with ACL rupture and a bar graph showing relative Caspase 3 protein expression normalized to β-actin in normal (*n* = 4) and ACL rupture chondrocytes (*n* = 6). (Error bars = standard errors, * *p* < 0.05). **c** and **d** Representative western blot of Caspase 3 expression in chondrocytes from patients with more than 18 months ACL injury compared to patients with ACL-injury up to 18 months and normal chondrocytes. A bar graph showing relative Caspase 3 protein expression normalized to β-actin in normal (*n* = 3), ACL-injury up to 18 months (*n* = 3) and ACL rupture more than 18 months chondrocytes (*n* = 3). (Error bars = standard errors, * *p* < 0.05 versus normal, NS ACL < 18 months versus ACL > 8 months)

To investigate the role of chondrocyte apoptosis in articular cartilage chondrocytes after ACL-injury, we evaluated caspase 3 protein expression levels and found a significant increase of caspase 3 expression in chondrocytes of patients with ACL-rupture compared to normal chondrocytes (Fig. 2a, b) (*p* < 0.05). No association was found between apoptosis and time of injury, as we observed no difference in caspase 3 expression in chondrocytes from patients with more than 18 months ACL injury compared to those that underwent surgery within the first 18 months after injury (Fig. 2c, d).

### IL-1β and IL-6 expression in ACL-deficient knees

**Fig. 3:**
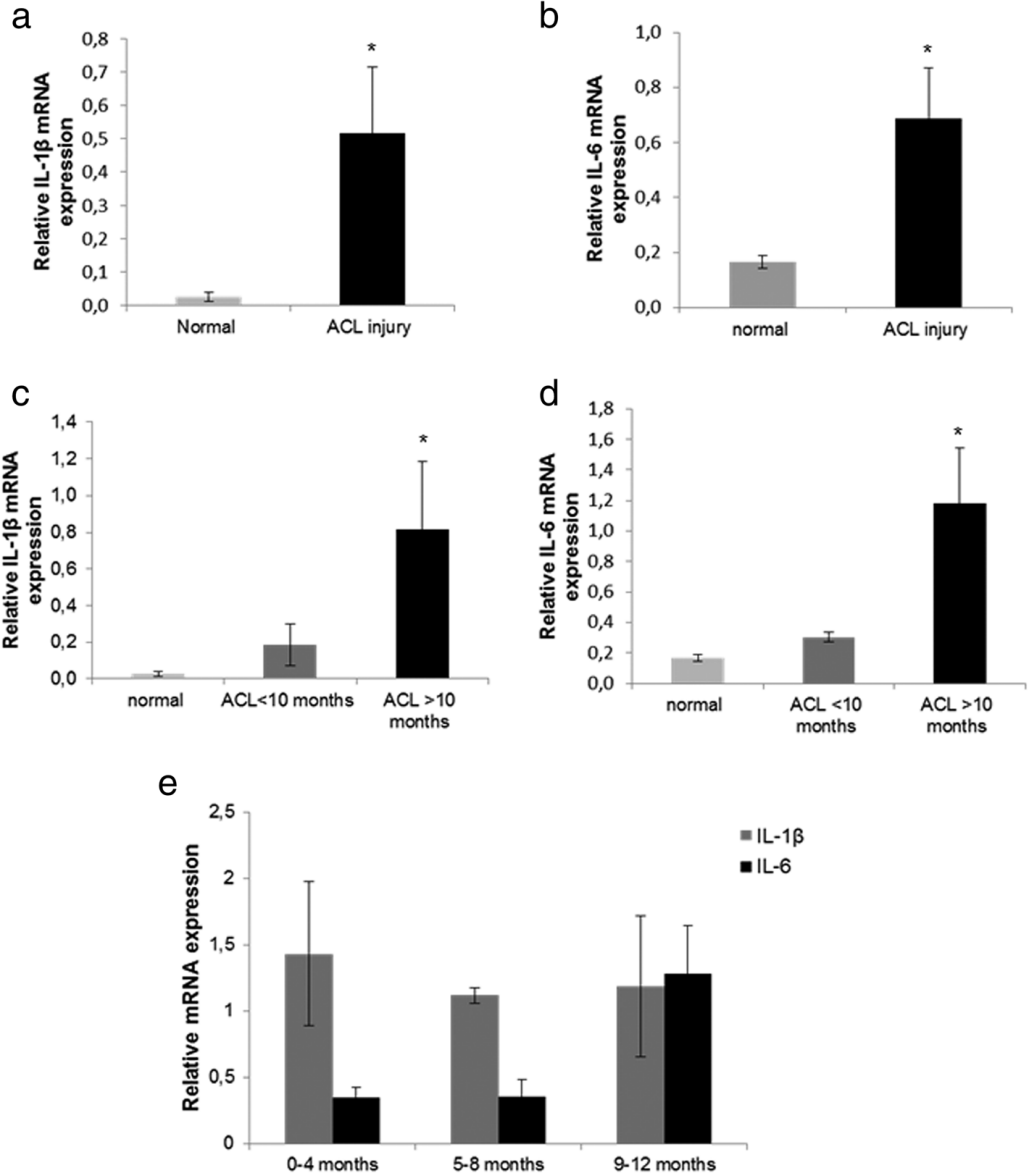
IL-1β and IL-6 expression in ACL-deficient knees. **a** and **b** Quantitative IL-1β and IL-6 mRNA expression in normal chondrocytes and chondrocytes from patients with ACL rupture (Error bars = standard errors, * *p* < 0.05). **c** and **d** Quantitative IL-1β and IL-6 mRNA expression in chondrocytes from patients with more than 10 months ACL injury compared to those that underwent surgery within the first 10 months after injury and normal chondrocytes (Error bars = standard errors, * *p* < 0.05). **e** Quantitative IL-1β and IL-6 mRNA expression in chondrocytes from patient with ACL rupture up to 12 months (group A: 1–4 months, group B: 5–8 months, and group C: 9–12 months

IL-1β and IL-6 mRNA expression levels were found to be upregulated in chondrocytes isolated from ACL-deficient knees compared to normal chondrocytes (Fig. 3a, b) (*p* < 0.05). Furthermore, we found an association between IL-1β and IL-6 mRNA expression levels and time course (time since trauma) after ACL injury, as we observed a significant upregulation of IL-1β and IL-6 expression in patients with ACL-rupture > 10 months from time of injury to arthroscopy compared to patients with ACL-injury up to 10 months (Fig. 3c, d) (*p* < 0.05). As IL-1β and IL-6 contribute to the acute inflammatory phase after ACL injury, the patient population with ACL rupture up to 12 months was divided into 3 groups: group A: 1-4 months (*n* = 8), group B: 5-8 months (*n* = 5), group C: 9–12 months (*n* = 3). Although there were no significant differences in IL-1β and IL-6 expression levels among the different groups, we observed a trend of increase of IL-6 expression in the 3^rd^ group (9–12 months) compared to the other groups (Fig. 3e). No association was found between IL-1β and IL-6 mRNA expression levels and cartilage defects (*p* = 0.899 IL-1β vs cartilage defects, *p* = 0.737 IL-6 vs cartilage defects) or patients’ age (*p* = 0.983 IL-1β vs patients’ age, *p* = 0.967 IL-6 vs patients’ age).

### MMP-13 expression in chondrocytes after ACL injury

**Fig. 4:**
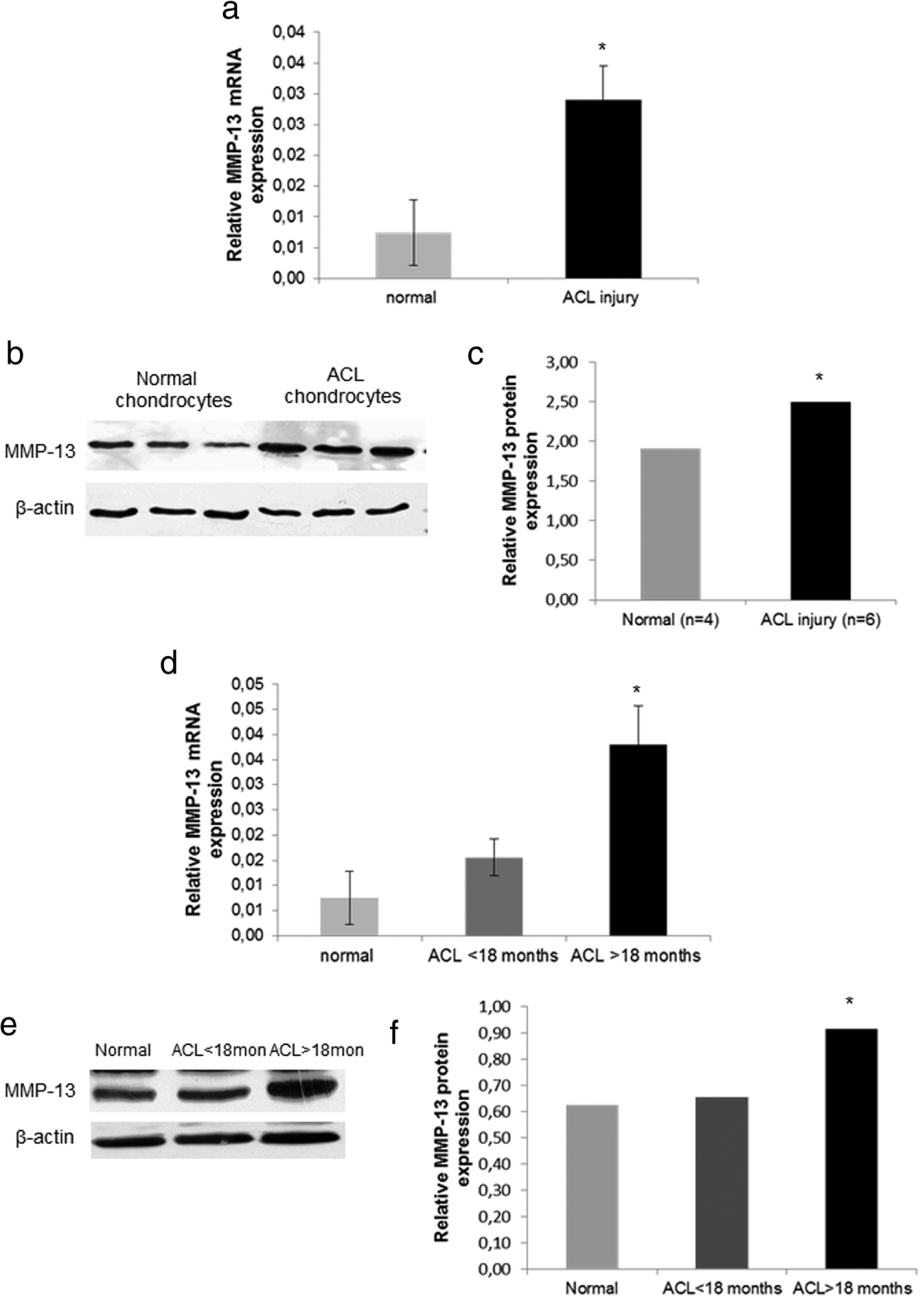
MMP-13 expression in chondrocytes after ACL injury. **a** Quantitative MMP-13 mRNA expression in cultured normal chondrocytes and chondrocytes from patients with ACL rupture. GAPDH was used for normalization of the real-time PCR data. (Error bars = standard errors, * *p* < 0.05). **b** and **c** Representative western blot of MMP-13 protein expression in cultured normal chondrocytes and chondrocytes from patients with ACL rupture and a bar graph showing relative MMP-13 protein expression normalized to β-actin in normal (*n* = 4) and ACL rupture chondrocytes (*n* = 6). (Error bars = standard errors, * *p* < 0.05). **d** Quantitative MMP-13 mRNA expression in chondrocytes from patients with more than 18 months ACL injury compared to those that underwent surgery within the first 18 months after injury and normal chondrocytes (Error bars = standard errors, * *p* < 0.05). **e** and **f** Representative western blot of MMP-13 expression in chondrocytes from patients with more than 18 months ACL injury compared to patients with ACL-injury up to 18 months and normal chondrocytes. A bar graph showing relative MMP-13 protein expression normalized to β-actin in normal (*n* = 3), ACL-injury up to 18 months (*n* = 3) and ACL rupture more than 18 months chondrocytes (*n* = 3). (Error bars = standard errors, * *p* < 0.05)

To provide evidence that extracellular matrix degradation observed after ACL injury is mediated by stimulation of catabolic enzymes, we evaluated MMP-13 expression levels in normal chondrocytes and in chondrocytes from patients with ACL rupture. We found significant upregulation of MMP-13 mRNA and protein expression levels in chondrocytes isolated from ACL-deficient knees compared to normal chondrocytes (Fig. 4a, b, c) (*p* < 0.05). Moreover, among patients with ACL-deficient knees, we observed significant upregulation of MMP-13 mRNA and protein expression in patients with ACL-rupture > 18 months from time of injury to arthroscopy compared to patients with ACL-injury up to 18 months (Fig. 4d, e) (*p* < 0.05). No association was found between ΜΜP-13 mRNA expression levels and cartilage defects (*p* = 0.900) or patients’ age (*p* = 0.306).

## Discussion

Although significant differences exist between patient populations from different ethnic origins followed in revision ACL cohorts [23], it is well established that both reconstruction and conservative treatment after ACL rupture are associated with increased risk for osteoarthritis development [4, 24, 25]. Biochemical changes in addition to altered joint biomechanics are among major contributing factors to post traumatic disease process [26], however, the mechanisms involved in articular cartilage degradation after injury are not well understood. In the present study, we sought to investigate molecular changes in chondrocytes indicative for cartilage degeneration and osteoarthritis development following ACL rupture.

We demonstrated a strong correlation between time from ACL injury to arthroscopy and severity of chondral damage. We observed that patients with more than two years ACL-injury presented chondral changes and high-grade cartilage lesions due to the long duration of knee instability, whereas no cartilage degeneration was observed in patients with ACL-injury up to 12 months, pointing to the influence of time from injury on osteoarthritic changes in the ACL deficient knee. This finding is in agreement with previous studies reporting an association among the progressive radiographic evidence of cartilage damage, the time of the reconstruction of ACL-injury and OA development in the ACL-deficient knee [27–31].

Joint instability after injury results in abnormal loading forces leading to changes in chondrocytes’ metabolism and cartilage degradation [32]. Chondrocytes’ apoptosis is considered to play an important role in articular cartilage disruption, as chondrocytes are responsible for maintaining the function and homeostasis of cartilage [33]. We demonstrated excessive activation of apoptotic mechanisms after chronic ACL injury evidenced by the increased levels of caspase 3; however, this activation was not associated with time from injury. Previous studies have identified chondrocyte death following impact to articular cartilage [34–36], while it has been shown that the superficial zone of cartilage is the most susceptible to cell death after mechanical injury [37]. Mechanical injury induces chondrocytes’ apoptosis and release of glycosaminoglycns (GAGs) from the matrix [38] suggesting the inhibition of progressive increase in apoptotic cells as a therapeutic target. However, whether chondrocytes’ apoptosis is a cause or a result of cartilage degeneration after injury and subsequent osteoarthritis is hotly contested.

It has been suggested that inflammatory cytokines are elevated in peripheral blood and/or synovial fluid following joint injury and that their levels may be correlated with severity of cartilage damage in osteoarthritis [15, 39–41]. To investigate the role of inflammatory cytokines in chondrocytes after ACL rupture, we evaluated IL-1β and IL-6 expression levels in chondrocytes isolated from patients with ACL-deficient knee and found significantly higher L-1β and IL-6 mRNA expression levels compared to normal chondrocytes. Moreover, we observed that differences in cytokine expression were correlated to time of injury, as IL-1β and IL-6 expression levels were higher in patients with more than 10 months ACL injury, suggesting the involvement of cytokines imbalance in the progressive cartilage degradation over time after ACL injury. Marks et al. demonstrated difference in cytokine profiles in synovial fluid between normal and injured knees and this difference varied based on the severity of chondral damage, which was associated with time from injury [15]. In addition previous studies demonstrated increased release of IL-1 and IL-6, in peripheral blood, as early as 1 to 6 h after surgery in the acute phase of inflammation [39, 40]. In acute anterior cruciate ligament injured knee the highest levels of inflammatory cytokines, IL-1β, IL-6, IL-8 and IL-10 were detected intra-articularly within few weeks or months after injury and subsequently all inflammatory cytokines decreased to levels observed in chronic arthritis [13, 42, 43]. This finding can explain why we did not observe differences in IL-1β and IL-6 expression levels in the group of patients with ACL deficiency up to 12 months, as patients in our study had subchronic and chronic ACL-injury and not acute. Moreover, a recent study demonstrated that two blockers of catabolic processes stimulated by cytokines, IGF-1 and Dex, inhibited the cytokine-mediated cartilage degradation in adult human cartilage providing a new approach for treatment of early- stage OA associated with joint injury [44].

The balance of tissue remodelling and synthesis is controlled by matrix metalloproteinases (MMPs) and their natural inhibitors (TIMPs) and it is widely accepted that the release of extracellular matrix-degrading enzymes is an important mechanism in post-traumatic cartilage damage [45]. We demonstrated significant upregulation of MMP-13 expression in injured chondrocytes and this upregulation was associated with time from injury, suggesting the contribution of the catabolic enzyme, MMP-13, in cartilage degradation during chronic ACL-injury. MMP-13 is a collagenase and has a predominant role in osteoarthritis due to its contribution to collagen degradation. MMP-13 also degrades the proteoglycan molecule, aggrecan, having a dual role in matrix destruction. Fragments of extracellular matrix that are generated by extracellular matrix-degrading enzymes stimulate further production of pathogenetic mediators [19], whereas chondrocytes have been demonstrated to express increased levels of MMP-1, MMP-3, MMP-8, MMP-9, MMP-13 and ADAMTS-5 after mechanical impact injury [46, 47]. In a recent study Haslauer et al. reported that upregulation of genes coding for proteins capable of degrading cartilage ECM, in adolescent minipigs, is observed within the first few days after ACL injury and this response is seen not only in chondrocytes, but also in cells in the synovium and ligament [48].

We must acknowledge a limitation of the present study, which is the small number of cartilage samples from individuals after ACL injury and from healthy ones.

## Conclusion

We demonstrated, for the first time, that chondrocytes after ACL injury produce apoptotic, inflammatory and catabolic factors and that increased levels of these factors are associated with time from injury contributing to cartilage degradation. Our data provide some indication that chondrocytes after ACL injury undergo molecular changes resembling those that take place in osteoarthritis, suggesting that factors responsible for articular cartilage homeostasis combined with appropriate surgical treatments may yield more effective therapies for post-traumatic osteoarthritis.

## Abbreviations

ACL: Anterior cruciate ligament
DMEM/F-12: Dulbecco’s modified eagles medium/ ham’s F-12
ECM: Extracellular matrix
FBS: Fetal bovine serume
IL-1β: Interleukin-1β
IL-6: Interleukin-6
MMP-13: Matrix metalloproteinase 13
PCR: Polymerase chain reaction
TIMP-1: Tissue inhibitor of metalloproteinases 1
